# The Impact of COVID-19 on Dental Treatment in Kuwait—A Retrospective Analysis from the Nation’s Largest Hospital

**DOI:** 10.3390/ijerph19159275

**Published:** 2022-07-29

**Authors:** Wasmiya Ali AlHayyan, Khalaf AlShammari, Falah AlAjmi, Sharat Chandra Pani

**Affiliations:** 1 Al Jahra Specialist Center, Al Jahra, Kuwait; dr_ali@hotmail.com (W.A.A.); falahalajmi@gmail.com (F.A.); 2 Faculty of Dentistry, Kuwait Institution for Medical Specialization, Kuwait City, Kuwait; kalsham71@gmail.com; 3 Schulich School of Medicine and Dentistry, University of Western Ontario, London, ON N6A 3K7, Canada

**Keywords:** access to dental care, COVID-19, dental public health

## Abstract

Background: The COVID-19 pandemic has changed the way dentistry has been practiced the world over. This study sought to assess the impact of the COVID-19 pandemic on the patterns of attendance for dental treatment in a large hospital in Kuwait through comparisons with data from the year prior to the pandemic. Methods: A total of 176,690 appointment records from 34,250 patients presenting to the AlJahra specialist hospital in Kuwait for dental treatment from April 2019 to March 2021 were analyzed. The types of procedures and the departments in which they presented were analyzed, and the patterns of attendance before and during the pandemic were compared. Results: While there was a significant reduction in the number of orthodontic, endodontic, and periodontal procedures, there were no impacts on oral surgery, restorative procedures, or pediatric dentistry. Conclusions: There has been a return in the number of patients obtaining dental treatment; however, there has been a definite shift in the use of certain dental procedures.

## 1. Introduction

The World Health Organization (WHO) announced the outbreak of a public health emergency of international concern on 30 January 2020, and a pandemic on 11 March 2020, affecting more than 7 million people in more than 188 countries [1]. The COVID-19 pandemic has changed the way dentistry has been practiced the world over [1,2,3,4]. The spread of the virus by aerosols has meant that dental practices the world over have had to find ways to contain aerosols in practices [5]. There is also data emerging showing that the pandemic has had different impacts on different dental specialties [5,6,7].

The impact of COVID-19 in Kuwait has been documented in the literature and the State adopted aggressive measures toward the containment of the pandemic, including an early and aggressive lockdown between 1 April and 30 May 2020. Between the declaration of the pandemic in March 2020 and the first administration of vaccines in April–May 2021, Kuwait saw variations in both the number of cases and mortality from COVID-19 [8]. There is, however, no data on how these factors affected the attendance of patients in dental clinics in Kuwait.

Dental care in Kuwait has been provided to all residents using a combination of subsidies and benefits since 1951. However, since 1992, growth and improvements in the economy have meant that dental care in Kuwait is provided by both government hospitals and private dental clinics [9]. While private clinics provide services for a fee, the government hospital provides free dental care to all who are eligible. Despite the growth of private dental care in Kuwait, the role of government centers in the provision of dental care and their impact on the overall well-being of the people of Kuwait has been documented in the literature [9,10].

The Kuwait dental administration at the ministry of health in Kuwait has built an integrated medical system based on recommended scientific policies and a clear methodology in the different dental specialties representing the largest medical specialties with regard to finance and human resources. A total of 1,083,272 patients benefit from dental services provided in polyclinics at distinct residential areas in Kuwait. The AlJahra Specialized Dental Center (ASDC) is the largest of the different governmental polyclinics, serving nearly 22.1% of the patients referred from polyclinics to specialized centers [10]. 

The ASDC is part of the new AlJahra hospital in the AlJahra governate, the largest governate among the six Kuwaiti governates, with the highest population density [9]. The ASDC serves the AlJahra governate and the surrounding AlJahra districts, with a population of 452,596 people [3]. Prior to the pandemic, this was one of the largest dental treatment centers in Kuwait, seeing over 20,000 patients per year. 

Data are emerging from around the world showing that the initial reluctance of individuals to seek dental treatment during the pandemic has been replaced by differing access to dental care [3,11,12,13]. While there have been some attempts to analyze data from multiple centers [14,15,16], there is little longitudinal data from a large public hospital. Newer variants of the virus and increased transmissibility mean the world has seen the pandemic slowly start to show features of an endemic. Data on how services were impacted by the COVID-19 pandemic are important for developing a better understanding of how dentists across the globe can deal with the challenges posed by this new phase of the disease. This study sought to assess the impact of the COVID-19 pandemic on patterns of attendance for dental treatment at the AlJahra Specialized Dental Center (ASDC) and compare them to data from the year prior to the pandemic.

## 2. Materials and Methods

### 2.1. Ethics Approval

Ethics approval for the study was obtained from the Standing Committee for the Co-ordination of Medical and Health Research, Ministry of Health, Government of Kuwait, to be carried out at AlJahra Hospital (1829/2021). All patients attending the hospital signed a form consenting to the use of their anonymized data for research purposes. The parents of children aged below 18 years signed this form on their behalf. 

### 2.2. Data Collection

The data were collected retrospectively from the patient management system (Patient Statistic Program-Microsoft Access 2000, Microsoft Corp., Palo Alto, CA, USA). Data regarding the age, gender, and nationality of the patients, as well as the department providing the treatment, were collected. Monthly COVID-19 case rates and vaccination rates for the AlJahra governate were obtained from the Central Statistical Bureau. The codes for each department were entered before each visit and were included in the data mining for the current study. The details of the appointment, the number of visits for each procedure, and the time taken per appointment were not mined for the current study.

### 2.3. Data Analysis

Patient data were exported using Microsoft Excel (Microsoft Corp., Palo Alto, CA, USA) and analyzed using the SPSS version 25 data processing software (IBM-SPSS, Armonk, NY, USA). Descriptive data were tabulated and the significance levels of differences among gender and nationality were calculated. Differences between pre-pandemic and pandemic levels of attendance were compared according to specialty, with differences calculated using the binomial test. Differences between the genders in each department were measured separately before and during the pandemic and tested for significance using the binomial test. All tests were carried out with a level of significance of *p* < 0.05. 

## 3. Results

**Figure 1:**
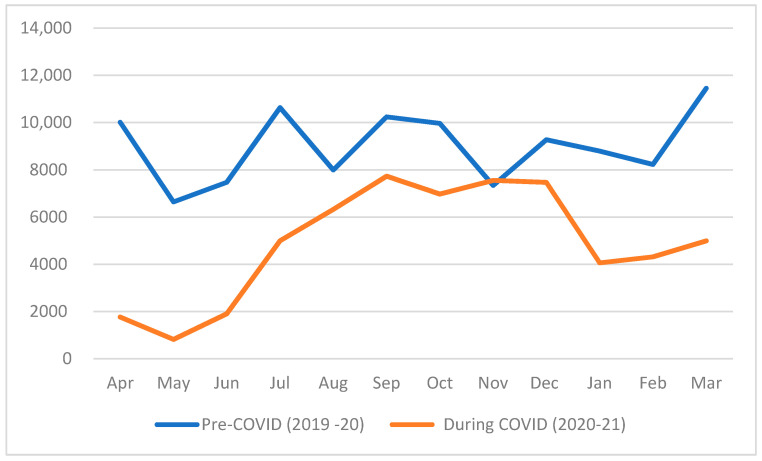
Impact of COVID-19 on overall patient presentation.

The sample was comprised of 176,690 records of appointment registration data from 34,250 patients who presented to the dental clinics of AlJahra hospital from March 2019 to March 2021. The sample was divided into two main groups: records of patients visiting before the pandemic (April 2019–March 2020) and during the pandemic (April 2020–March 2021) (Figure 1). Overall, there were fewer appointments during the pandemic (*n =* 83,813) when compared to the previous year (*n =* 92,598). A description of the mined data is presented in Table 1.

When divided according to gender, it was observed that there were significantly more female patients seen in all departments except for the COVID-19 unit and the pediatric dentistry department. This trend was the same before and during the pandemic (Table 2).

The impact of the pandemic was compared by specialty (Table 3). It was observed that different specialties were affected differently. While there was a significant reduction in patients seen in orthodontics, endodontics, and periodontics, no significant reductions were observed in prosthodontics. In both pediatric dentistry and oral surgery, there was an increase in the number of patients seen, although the differences were not statistically significant.

**Figure 2:**
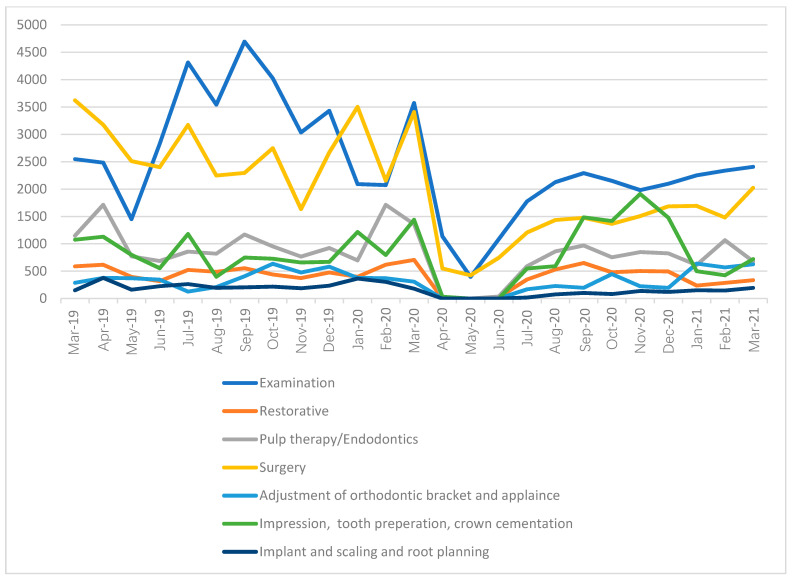
The number of treatments rendered before and during the pandemic, by type of treatment.

When the types of treatment rendered were tabulated (Figure 2), it was observed that all types of procedures were impacted by the first shutdown from April 2020 to June 2020. During this period, almost no aerosol-generating procedures were performed (Table 4). However, after the lifting of restrictions, a rebound in the number of procedures was observed (Figure 2). It was observed that variations were greatest among procedures such as prosthodontics, restorative procedures, and orthodontics (Figure 2).

## 4. Discussion

It has now been over two years since the first COVID-19 case was diagnosed, and the impacts on dentistry are becoming clearer [14,15,17,18]. This study aimed to follow the patterns of patient care over a one-year period from the implementation of the first COVID-19 restrictions in Kuwait. As data emerge on the ways that dental practices are adapting to pandemic-induced restrictions globally, this study sought to visualize the changing patterns in a large governmental hospital as the pandemic progressed.

The initial stages of the pandemic, from April 2020 to July 2020, were periods of effective shutdown for dental practices across the globe [8]. The lack of clear guidelines on the risks of aerosols, combined with global shutdowns and/or lockdowns, meant that elective dental procedures were not performed [4,18,19]; this is reflected in the drop in cases seen between April and July 2020. As restrictions on aerosol-generating procedures were gradually lifted, there was a slow increase in the number of patients seen. Our data show that between June and July 2020 there was a sharp increase in the number of patients seen for examination and surgery. This is explained by the fact that as operatories were allowed to perform aerosol-generating procedures, emergency procedures were re-prioritized. This is also in keeping with studies that showed that emergency procedures in the early days of the pandemic were restricted to extractions [4,17].

The increase in restorative and endodontic procedures during the period from July 2020 to the end of the study is of great significance. Our data suggest that there was a gradual increase in the number of these procedures being performed. The data also show that once these procedures were being performed, there was little variation in the number of procedures. This suggests that endodontic and restorative procedures are essential to the well-being of individuals. The tendency to restrict emergency procedures to extractions in the early days of the pandemic was based largely on the fact that there were insufficient operatories to manage aerosol-generating procedures [4,19,20]. The pandemic has resulted in the re-designing of dental operatories, both in small practices and large hospitals [2,3,5,19,21]. The data from this study show that the availability of sufficient rooms to perform these procedures is essential for the delivery of optimal dental care. The data also show that the hospital was able to create an infrastructure that could support the practice of aerosol-generating procedures. In this case, the hospital was a public organization, and funding from the government allowed for the creation of the necessary infrastructure. The financial toll on practices both large and small is beyond the scope of the current paper but is an important area for further research.

The fact that there were variations in orthodontic procedures suggests that these procedures are perhaps viewed as non-essential. While an argument can be made for these procedures to be provided a lower priority than pain-relieving procedures, such as endodontics, the impacts of treatment suspension on the outcomes of tertiary dental care are emerging [22,23]. Literature shows that prolonging or postponing orthodontic care that has already started can result in care being extended for long periods, with adverse outcomes, not only for orthodontic outcomes but also for oral hygiene [24].

The current dataset used secondary data, and since the operatory used for the provision of ultrasonic scaling is the same as the operatory used for the placement of implants, the data on the numbers of these procedures are presented together. The results are worrisome, as they show a significantly lower number of procedures when compared to restorative or endodontic care. Good ultrasonic scaling has long been viewed as the bedrock of dental hygiene maintenance [7]. However, the restrictions imposed on aerosol-generating procedures, have meant that, across the world, dentists have had to either limit or altogether stop the number of ultrasonic scaling procedures [5,7,14,15,18]. The results of the current study show that while the rates of endodontic and restorative procedures classified as “essential” have returned to pre-pandemic levels, the rates of scaling/root-planing/implant procedures are at half the pre-pandemic levels. 

Since the pandemic began, research has focused on both minimizing aerosols in dental practices and the optimal allocation of rooms where aerosol-generating procedures can be safely carried out [15,16,17]. The results of this study highlight the fact that pain-relieving procedures, such as restorative care, oral surgery, and endodontics quickly bounced back to pre-pandemic levels. A more interesting find is that while the placement of orthodontic brackets had returned to pre-pandemic levels by March 2021, ultrasonic scaling procedures had not. This finding mirrors global trends that show that while non-aerosol generating procedures quickly return to pre-pandemic levels, practices (both large and small) struggle to create new infrastructure to cope with the requirements for aerosol-generating procedures [12,17]. Orthodontic care is known to be associated with poorer oral hygiene outcomes [21]. The absence of or limitations in access to good ultrasonic scaling can have potential adverse effects that need to be addressed. 

The results of this study have to be viewed keeping in mind the overall changes in dentistry, both globally and within the Middle Eastern region [25,26]. The results of this study are in keeping with those of Cha and Cohen, who showed that there were significantly fewer adults who received a dental check-up in 2019 compared to 2020 [25]. The practice of dentistry in large hospital-based or hospital-like situations differs in many ways from the practice in a smaller individual or group dental practice. Since the beginning of the pandemic, there have been several factors that have affected the practice of dentistry in hospital settings. The fact that the pattern of practices changed during the pandemic has been previously documented. However, our results suggest that even after the lifting of restrictions there are certain shifts in the practice of dentistry that have continued to remain in place. This is in keeping with other hospital data from the region, which suggested changes in dental practice in a hospital setting in Saudi Arabia [27]. It is, therefore, reasonable to assume that large hospitals in the region will need to further evaluate the impact these changes have on the cost of care, the efficiency of care delivery, and the impact of these changes on patient satisfaction.

There are certain limitations of this study. The study only looked at practices in a hospital setting and does not reflect the challenges faced by smaller practices. Furthermore, the scope of the current study was only the overall pattern of attendance of the patients. The actual treatment rendered and the number of appointments per procedure were not recorded and were beyond the scope of this study. Despite these limitations, the study has many strengths, including the large sample size and the fact that the study followed the population into 2021 to fully visualize the longer-term impacts of COVID-19-related changes on the practice of dentistry.

## 5. Conclusions

The results of this study show that while attendance in dental clinics at the AlJahra hospital has nearly returned to pre-pandemic levels, there have been significant shifts in the types of procedures performed. The long-term impacts of these shifts are deserving of future research in order to provide comprehensive dental care to patients as well as to better plan for future waves of the pandemic.

## Figures and Tables

**Table 1:** Demographic profile of the population.

	Gender	Age Group			
		Below 12 Years	13–44 Years	45–64 Years	Above 65 Years
No. of Patients	Male	1123	8113	3101	883
	Female	1213	12,677	6234	906
No. of Appointments	Male	15,605	40,391	11,667	2219
	Female	16,784	69,027	18,583	2414

**Table 2:** Attendance differences according to gender.

	Department	Gender				*p* *
		Male		Female		
		Count	Row N %	Count	Row N %	
During the Pandemic (2020–2021)	Pediatric Dentistry	7162	47.9%	7786	52.1%	0.187
	COVID-19 Unit	N/A	N/A	N/A	N/A	N/A
	Prosthodontics/Operative	5270	37.0%	8958	63.0%	<0.001 **
	Orthodontics	2717	30.2%	6270	69.8%	<0.001 **
	Oral Surgery	12,880	41.7%	18,032	58.3%	<0.001 **
	Endodontics	3831	38.2%	6204	61.8%	<0.001 **
	Periodontics	1645	35.0%	3054	65.0%	<0.001 **
Pre-Pandemic (2019–2020)	Pediatric Dentistry	7073	48.0%	7664	52.0%	0.865
	COVID-19 Unit	338	49.3%	347	50.7%	0.906
	Prosthodontics/Operative	5026	33.4%	10,030	66.6%	<0.001 **
	Orthodontics	3576	33.7%	7027	66.3%	<0.001 **
	Oral Surgery	11,253	40.5%	16,519	59.5%	<0.001 **
	Endodontics	6247	37.9%	10,253	62.1%	<0.001 **
	Periodontics	2659	36.7%	4585	63.3%	<0.001 **

* Calculated using the binomial test. ** Differences significant at *p* < 0.05.

**Table 3:** Impact of the pandemic on attendance by specialty.

	During the Pandemic (2020–2021)	Pre-Pandemic (2019–2020)	*p* *
Pediatric Dentistry	14,948	14,737	0.564
COVID-19 Unit	NA	685	NA
Prosthodontic/Operative	14,228	15,056	0.148
Orthodontics	8987	10,603	0.021 **
Oral Surgery	30,912	27,772	0.076
Endodontics	10,035	16,500	0.005 **
Periodontics	4699	7244	0.001 **

* Calculated using the binomial test. ** Differences significant at *p* < 0.05.

**Table 4:** Type of procedure performed by month from March 2019 to March 2021.

	Examination	Restorative	Pulp Therapy/Endodontics	Surgery	Adjustment of Orthodontic Bracket and Appliance	Impression, Tooth Preparation, Crown Cementation	Implant, Scaling, and Root Planning
Mar-19	2547	589	1149	3621	288	1075	151
Apr-19	2484	618	1712	3175	378	1130	379
May-19	1450	397	771	2511	371	799	163
Jun-19	2827	318	686	2402	345	555	226
Jul-19	4313	524	859	3172	128	1180	265
Aug-19	3543	490	820	2248	210	399	195
Sep-19	4694	556	1170	2295	408	748	205
Oct-19	4025	440	953	2748	635	727	218
Nov-19	3035	374	768	1634	477	660	188
Dec-19	3431	477	925	2670	582	670	235
Jan-20	2090	399	697	3502	378	1216	365
Feb-20	2073	620	1712	2155	371	796	305
Mar-20	3574	706	1358	3409	309	1441	181
Apr-20	1137	4	5	550	0	35	0
May-20	397	0	0	426	0	1	0
Jun-20	1088	28	38	747	0	0	0
Jul-20	1777	349	591	1208	170	550	19
Aug-20	2128	531	862	1435	231	592	77
Sep-20	2292	651	970	1475	197	1484	102
Oct-20	2151	481	755	1366	448	1417	83
Nov-20	1981	503	849	1506	225	1912	139
Dec-20	2098	495	826	1683	195	1478	120
Jan-21	2251	238	614	1693	640	500	152
Feb-21	2338	287	1067	1481	570	426	146
Mar-21	2406	337	680	2024	627	721	195

## Data Availability

Data will be made available upon reasonable request to the authors.
